# Quality control in the initiation of eukaryotic DNA replication

**DOI:** 10.1098/rstb.2011.0073

**Published:** 2011-12-27

**Authors:** John F. X. Diffley

**Affiliations:** Cancer Research UK London Research Institute, Clare Hall Laboratories, South Mimms, Herts EN6 3LD, UK

**Keywords:** DNA replication, cell cycle, S phase

## Abstract

Origins of DNA replication must be regulated to ensure that the entire genome is replicated precisely once in each cell cycle. In human cells, this requires that tens of thousands of replication origins are activated exactly once per cell cycle. Failure to do so can lead to cell death or genome rearrangements such as those associated with cancer. Systems ensuring efficient initiation of replication, while also providing a robust block to re-initiation, play a crucial role in genome stability. In this review, I will discuss some of the strategies used by cells to ensure once per cell cycle replication and provide a quantitative framework to evaluate the relative importance and efficiency of individual pathways involved in this regulation.

## 1. Introduction

The genomes of eukaryotic cells are replicated from many replication origins distributed along multiple chromosomes during the synthetic or S phase of the cell cycle. This parallel processing approach to tackling DNA replication was significant in the evolution of eukaryotes; it allows cells to replicate even very large genomes in relatively short periods of time, and was, therefore, crucial in supporting the increase in genome size needed for complex multi-cellular life. However, this required the solution to a serious bookkeeping problem: the cell must ensure that sufficient numbers of origins are used in each S phase without the re-use of any origin in a single cell cycle. The use of an insufficient number of origins would leave regions of the genome unreplicated before mitosis, generating broken chromosomes and loss of genetic information, while re-initiation from any origin would lead to unbalanced increases in gene dosage and long-lived, potentially fragile replication forks. In this review, I will describe the basic mechanisms that ensure once per cell cycle replication in eukaryotic cells and explore some of the complexities in this regulation. I will also provide some quantitative arguments to explain why this complexity exists. The reader is referred to a number of excellent recent reviews for further detail [1–8].

## 2. Design of the system

In bacterial and eukaryotic cells, a group of related ‘initiator’ proteins specifies where replication origins will be located and then act to load hexameric DNA helicases required to unwind DNA during DNA replication. There is a fundamental difference in the way the helicases are loaded in these two systems and it is this difference that is key to understanding the control of eukaryotic replication [7]. In the Gram-negative bacterium *Escherichia coli*, the dnaA protein acts as the ‘initiator’ protein: it binds as an oligomeric filament to multiple specific sequences within the origin of replication (oriC) and dictates where DNA unwinding, and hence replication, will begin [9,10]. dnaA is a member of the AAA+ family of ATPases, and ATP plays a crucial role in dnaA function [11]. Although dnaA can bind to oriC without ATP, only the ATP-bound form of dnaA can induce DNA melting in a region adjacent to the dnaA-binding sites known as the 13mers. dnaA, together with another AAA+ protein dnaC, then loads one hexameric helicase (dnaB) around each of the single strands of the melted 13mers. Once the helicases are loaded, they can begin to unwind DNA and bidirectional replisomes can be assembled on the unwound DNA.

In *E. coli*, the first step in replication, dnaA binding and origin melting, is tightly regulated by a variety of mechanisms that are critical for preventing the immediate re-initiation of replication (see [9,10] for further discussion). Some mechanisms regulate the occupancy of oriC by dnaA (e.g. SeqA, DatA) and some regulate the nucleotide state of dnaA (RIDA, DARS, β-clamp). Although a detailed description of replication control in bacteria is beyond the scope of this review, a few points are relevant and worth making. Firstly, as we will see below, the use of multiple mechanisms to prevent re-replication is also a feature of eukaryotic DNA replication. Secondly, many of the mechanisms involved in preventing re-replication are not conserved between different bacterial species. For example, in *E. coli*, methylation of adenine residues in GATC sequences by Dam methylase plays a critical role in preventing re-replication [12,13]. Before replication, oriC is fully methylated by Dam methylase, but immediately after replication, because the nascent DNA strand is unmethylated, the double-stranded origin is transiently hemi-methylated before Dam methylase can methylate the nascent DNA. The SeqA protein binds tightly to this hemi-methylated DNA, preventing dnaA from re-binding to its weaker binding sites, thus preventing re-initiation of replication [13–15]. Although the Dam/seqA system is clearly important in *E. coli* and mutants lacking this system inappropriately re-initiate replication, this system is entirely absent from other bacterial groups such as the Gram-positive bacteria including *Bacillus subtilis*. Again, as we will see below, the apparently rapid evolution of re-replication control is also a feature of eukaryotic replication.

**Figure 1.**
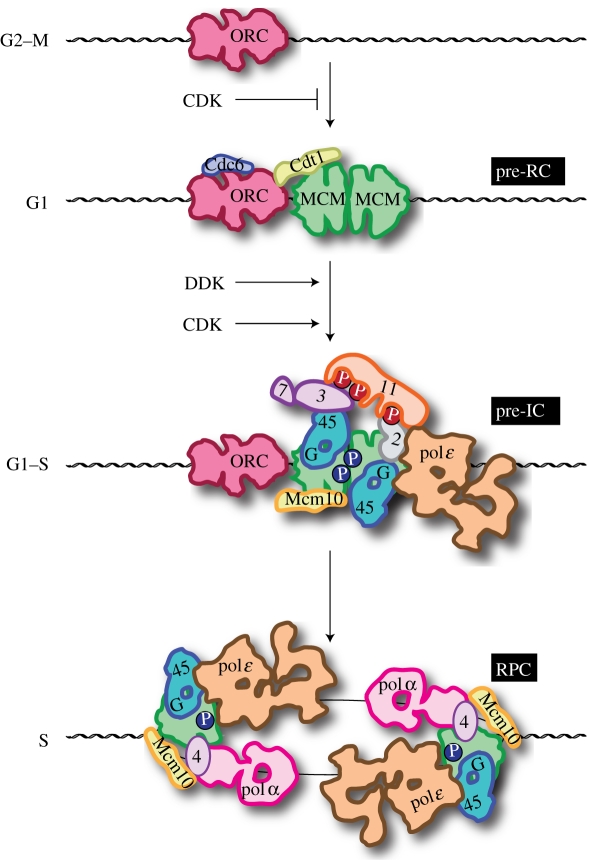
Stepwise assembly of DNA replication complexes. The individual steps leading to the assembly of bidirectional replisomes is outlined. Names associated with each of the complexes are shown on the right: pre-RC, pre-replication complex; pre-IC, pre-initiation complex; RPC, replisome progression complex. Cell cycle phases permissive for the individual steps are shown on the left. For simplicity, some of the protein names have been abbreviated: 11, Dpb11; 3, Sld3; 7, Sld7; 2, Sld2; G, GINS; 45, Cdc45; 4, Ctf4. The shapes of many of the individual components are loosely based on three-dimensional reconstructions from electron micrographs: ORC and Cdc6 are from Chen *et al*. [17], Mcm double hexamer is from Remus *et al*. [18], Cdc45, Mcm2-7, GINS (CMG) are from Costa *et al*. [19], DNA polymerase epsilon (pol*ɛ*) is from earlier studies [20,21], and DNA polymerase α (polα) is from Klinge *et al*. [21]. The roles of CTF4 and Mcm10 in the RPC are inferred from earlier studies [22,23]. Cyclin-dependent kinase (CDK) phosphorylations are shown in red, Dbf4-dependent kinase (DDK) phosphorylations are shown in blue. The order of DDK and CDK in activating replication comes from earlier studies [24–26].

In eukaryotes, the initiator protein is a multi-subunit protein called the origin recognition complex (ORC; figure 1) [16]. Five of the six ORC subunits are members of the AAA+ family (though only one, Orc1, has retained a functional ATPase [27]) in a clade of initiator proteins that includes dnaA [28]. Analogous to cooperation of dnaA with the AAA+ dnaC, ORC cooperates with another AAA+ ATPase, Cdc6, to load the replicative helicase, Mcm2-7, onto origin DNA [29,30]. An additional factor, Cdt1, which does not have an obvious bacterial analogue, is also essential for helicase loading [31–33]. ATP also plays a crucial role in ORC function; however, this role is quite different from the role of ATP in dnaA function. ATP binding (but not hydrolysis) is required for budding yeast ORC to bind to its cognate sequence within origins; ADP cannot fulfil this requirement [16]. In contrast to dnaA, the binding of ORC to origin DNA does not induce measurable origin melting in the region of helicase loading. Whereas ATP hydrolysis by dnaA primarily plays a regulatory role by preventing immediate re-initiation, ATP hydrolysis by ORC and Cdc6 is required for the loading of the hexameric Mcm2-7 helicase onto DNA [34,35].

The most important difference with bacteria derives from this fact that ORC does not melt DNA. Instead, it loads Mcm2-7 as a head-to-head double hexamer around double-stranded DNA, not single-stranded DNA [18,36]. In this configuration, in contrast to the situation in bacteria, the helicase is inactive. Indeed, this pre-replicative complex (pre-RC) can reside at origins for significant periods of time before the helicase is activated and DNA replication commences [37]. Activation of the helicase is a complex and still poorly characterized step that involves the reconfiguration of Mcm2-7 so that the double hexamer separates and the individual hexamers are bound around each of the single strands of DNA [2,38,39]. This step requires the loading of two additional proteins, Cdc45 and GINS, onto Mcm2-7 in a reaction requiring another set of factors including Sld2, Sld3, Sld7 and Dpb11 [2]. The Cdc45–Mcm2-7–GINS complex then acts as the replicative helicase [40], where it is essential for DNA unwinding during the elongation phase of DNA replication [41–46].

The division of the initiation reaction into two discrete steps (helicase loading and helicase activation) in eukaryotes has profound implications for the control of DNA replication [7]. By differentially regulating these two steps so that they are restricted to different phases of the cell cycle, cells can ‘license’ hundreds or thousands of origins with the inactive helicase, and these origins can subsequently be ‘fired’ by helicase activation. Because the helicase cannot be reloaded in S phase, origin firing is limited to once per cell cycle. In general, licensing can occur from the end of mitosis until a point in late G1 phase, and the helicase activation step can only occur during S phase and G2 phase. The mechanisms by which these steps are regulated during the cell cycle are considered in §3.

## 3. Regulation of licensing

Pre-RC assembly is restricted to late M/early G1 phase by at least three separate systems in eukaryotes. In some organisms, all three are used, whereas in others only one or two. These systems include geminin-dependent inhibition of Cdt1 function, Cul4 (Crl4)- and proliferating cell nuclear antigen (PCNA)-dependent degradation of Cdt1 during S phase and cyclin-dependent kinase (CDK)-dependent inhibition of licensing via a variety of targets. These have been the subject of a number of very thorough reviews and the reader is encouraged to consult these for more detail [1–4,6–8,47–49].

Geminin is a small, coiled-coil protein originally identified in *Xenopus* egg extracts as a substrate for the anaphase-promoting complex/cyclosome (APC/C) [50] and an inhibitor of Cdt1 function in licensing [51,52]. Because the APC/C is specifically active during mitosis and G1 phase, geminin is inactivated during this period, allowing Cdt1 to participate in the licensing reaction only during this period. Geminin appears to be found only in metazoans, where it contributes to preventing re-replication. Depletion or deletion of geminin induces a G2/M checkpoint in many different cell types. In some cell types, this is accompanied by substantial re-replication, whereas in other cell types it is accompanied by S phase delays. Although these phenotypes appear superficially contradictory, they probably derive from the same root cause: induction of re-replication. In some cases, large numbers of origins are deregulated, resulting in significant amounts of re-replication. In others, only a few origins may be deregulated resulting in very small amounts of re-replication [53]. In some cases, this may even be manifested as an apparent reduction in overall amounts of DNA replication [53] presumably because checkpoint activation caused by re-replication can prevent all new initiation, leading to an overall shut down of replication. In general, cancer cells appear especially prone to re-replication after geminin depletion [54,55].

The second system contributing to preventing re-replication involves the targeting of Cdt1 for degradation during S phase by an E3 ubiquitin ligase containing Cul4 (Crl4), Ddb1 and Rbx1 and using the Cdt2 substrate recognition subunit [56–58]. In this system, Cdt1 is recruited to chromatin specifically during S phase by interaction with the PCNA sliding clamp processivity factor where it is ubiquitylated and destroyed [59,60]. This system elegantly couples the prevention of re-replication directly to the act of replication and, as a consequence, operates only during S phase of a normal cell cycle. Cul4-dependent Cdt1 degradation has been conserved from fission yeast through metazoans [61,62].

The final system working in most eukaryotes is the only system operative in the budding yeast *Saccharomyces cerevisiae*. This system involves direct inhibition of pre-RC components by CDKs (reviewed in [63]). Because CDKs are inactivated at the end of mitosis and become re-activated in the late G1 phase, this establishes a window of time during G1 phase when licensing can occur. In budding yeast, where this has been best characterized, Cdc6, ORC and Mcm2-7 are all directly inhibited by CDK phosphorylation, each by different mechanisms. The Cdc6 protein is phosphorylated by both the G1 phase cyclin (CLN)-associated CDK as well as the S/G2/M phase cyclin (CLB)-associated CDK [64–67]. CDK phosphorylation of Cdc6 generates two distinct binding sites for the Cdc4 subunit of the Skp, Cullin, F-box containing complex (SCF) ubiquitin ligase [66]. Thus, CDK phosphorylation of Cdc6 inhibits its function by targeting it for ubiquitin-mediated proteolysis. Later in the cell cycle, the mitotic cyclin Clb2 binds tightly to CDK-phosphorylated Cdc6 and prevents it from associating with ORC [68]. The Clb2-binding site overlaps one of the SCF-binding sites, and stabilizes the Cdc6 protein in an inactive form [66,68]. The degradation of Clb2 at the very end of mitosis by the APC/C releases Cdc6 from its inhibitory complex and allows licensing to occur. CDKs inhibit the Cdt1/Mcm2-7 complex by promoting its export from the nucleus [69,70]. This, again, is accomplished by both CLN- and CLB-associated CDK [70] and involves direct phosphorylation of Mcm subunits [71]. Finally, ORC is phosphorylated on two subunits (Orc2 and Orc6) specifically by the CLB-associated CDK [72,73]. ORC phosphorylation inhibits pre-RC assembly by interfering with the interaction between ORC and Cdt1 [74].

In addition to this role in inhibiting pre-RC assembly, CDKs play a second essential role in regulating DNA replication: they are required to trigger initiation from licensed origins. They do this in budding yeast by phosphorylating Sld2 and Sld3 [75–77]. Phosphorylation of these proteins generates binding sites for tandem BRCT repeats in the Dpb11 protein. Recently, it has been shown that essential CDK phosphorylation has been conserved in the human homologue of Sld3, Treslin/ticrr [78,79]. As a consequence of these two distinct roles for CDKs, pre-RC assembly is restricted to G1 phase, when origins cannot fire because CDKs are absent, and activation of CDK at the end of G1 phase triggers initiation from licensed origins, and prevents the re-assembly of pre-RCs at origins that have fired.

The role for CDK in preventing licensing outside of G1 phase has been conserved in evolution; however, the specific details of how CDK inhibits licensing are quite different in different organisms. Chemical inhibition of CDKs in G2 and mitosis or genetic depletion of the mitotic CDK1 promote re-licensing and allow additional rounds of replication in human tissue culture cells [80–82], very similar to re-replication induced by deletion of mitotic cyclins in fission yeast [83] and transient inhibition of CDK by overexpression of CDK inhibitors in both fission and budding yeasts [84,85]. Moreover, in at least some cell types, cyclin A depletion increases the amount of re-replication caused by geminin depletion [86]. Some targets of CDK inhibition in human cells have been identified: both Orc1 and Cdt1 can be targeted for SCF^Skp2^-dependent degradation [59,87] and Cdc6 phosphorylation can cause its export from the nucleus [88–91]. Because of the importance of geminin and Crl4-dependent Cdt1 degradation and because these pathways do not exist in budding yeast, regulation of pre-RCs by CDKs in metazoans has been somewhat understudied, and further work is required to understand its importance relative to the other two pathways.

Given the importance of preventing re-initiation of DNA replication within a single cell cycle, it might seem odd that details of this critical mechanism have not been conserved in evolution. It is likely that two factors contribute to the rapid evolution of licensing regulation by CDKs. The first is the high level of overlap built into the system. For example, while Cdc6 is well established as a CDK target in budding yeast, mutation of any individual phosphorylation site in Cdc6 does not induce re-replication or result in significant reduction in fitness. Indeed, a version of Cdc6 in which all CDK sites have been eliminated is viable, grows apparently normally and does not show defects in origin function as indicated by plasmid maintenance [64]. Similarly, forced nuclear localization of Mcm2-7 throughout the cell cycle or expression of unphosphorylatable *orc* mutants alone does not induce detectable re-replication even using sensitive comparative genome hybridization methods [92]. It is only when deregulated components are combined that detectable re-replication occurs. For example, expression of stabilized Cdc6 together with unphosphorylatable ORC is lethal and induces re-initiation from a subset of replication origins [68,72,92]. It is only when all three proteins are deregulated that substantial amounts of DNA re-replication can be detected, for example, by flow cytometry [73].

The second factor that contributes to rapid evolution is the interchangeability of regulatory mechanisms [93]. For example, although combination of stable Cdc6 with an unphosphorylatable ORC is lethal, this lethality is suppressed by fusion of a cell cycle-dependent degron onto the Cdt1 protein, which confers CDK-dependent degradation of Cdt1 during S, G2 and M phases [93]. Also, addition of a cassette that confers CDK-dependent nuclear export onto stable Cdc6 is sufficient to restore viability when combined with unphosphorylatable ORC [93]. Thus, it appears that the molecular mechanisms by which each pre-RC component is inhibited by CDK are relatively unimportant; what is important is that multiple pre-RC components are inhibited by different mechanisms.

## 4. The quality control problem

To understand why so many mechanisms are involved in preventing re-initiation, it is useful to consider the scale of the problem: in cells with large genomes, such as humans, re-initiation needs to be prevented at tens of thousands of replication origins in each cell cycle over the course of billions of cell cycles. Thus, the block to re-replication needs to be extraordinarily efficient. In the following section, I will examine the implications this scale has on the problem. This was discussed in further detail in a previous review [94]. I will initially consider the issue in budding yeast, making a few simple assumptions. Firstly, DNA replication in yeast initiates from approximately 400 origins during each S phase, and re-initiation from any of these origins counts as re-initiation. In human cells, the number is approximately 50 000. Secondly, although the probability of re-initiating DNA replication is a function of both the probability of re-licensing origins and the probability of firing these re-licensed origins, for simplicity, we will set this second probability to ‘1’ (i.e. any origin inappropriately re-licensed will re-initiate). Therefore, re-replication is entirely a function of inappropriate re-licensing. Thirdly, each origin acts independently. That is, each origin has some probability of re-initiating that is unaffected by events at other origins.

With these assumptions, the probability that any individual origin will re-initiate in a single cell cycle can be converted into a probability that at least one origin in the genome will re-initiate as follows: if *p* is the probability an individual origin will re-initiate in one cell cycle, *q* is the probability an individual origin will not re-fire in one cell cycle and *n* is the number of origins (400 in yeast), then:
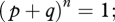
expanding this yields:
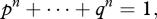
where *p*^*n*^ is the probability that all origins will re-fire while *q*^*n*^ is the probability that no origin will re-fire in a single cell cycle. All intermediate terms (not shown) are the probabilities of different numbers of origins re-firing. Thus, if the probability that a single origin will not re-fire in a cell cycle is 99 per cent (0.99), that is, the block to re-initiation per origin is 99 per cent efficient in a single cell cycle, then the probability that no origin will re-fire in yeast is (0.99)^400^ = 0.018, or approximately 2 per cent. In human cells, this is (0.99)^50 000^ = 6 × 10^−219^! Thus, to achieve a robust block to re-replication in each cell cycle, the block to re-initiation on a per origin basis must be far, far greater than 99 per cent. To achieve a 99 per cent probability that no origin will re-fire in a single cell cycle in yeast, the probability on a per origin basis is 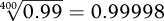, or 99.998 per cent efficient. In human cells, this is 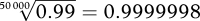. Or, in other words, an error rate of approximately 1 in 10^5^ initiation events in yeast and approximately 1 in 10^7^ in human cells is required to achieve this 99 per cent probability. Given the fact that even very limited re-replication is lethal in yeast [68,72,92], it is highly likely that the overall block to re-initiation in wild-type cells is considerably greater than 99 per cent.

## 5. A possible solution

From the preceding discussion, it is clear that the effective error rate in preventing re-initiation is likely to be even lower than 10^−7^ per origin. This approaches the kinds of error rates observed in nucleotide insertion during DNA replication, and it is worth comparing the systems. In the case of nucleotide incorporation, accuracy is achieved by a series of sequentially acting biochemical quality control mechanisms: replicative DNA polymerases have very high levels of accuracy in initial incorporation, they also have additional ‘proofreading’ exonucleases that can remove misincorporated bases immediately after insertion, and mismatch repair can catch any misincorporation that slips through these first two mechanisms [95]. In the prevention of re-initiation, mechanisms do not appear to act sequentially, but rather act in parallel. Nonetheless, the outcome is similar: extraordinary accuracy.

To understand how parallel mechanisms cooperate quantitatively, we assume that each mechanism operates independently of the other mechanisms. Using budding yeast as an example, this means that Cdc6 degradation is independent of Mcm2-7 nuclear export, which, in turn, is independent of ORC phosphorylation, etc. Importantly, re-initiation will only occur at an origin if all mechanisms fail. So, if we consider three separate mechanisms, each with a probability of failing (*p*_Cdc6_, *p*_ORC_, *p*_Mcm_), then the probability that all three will fail (*p*_all_) is:
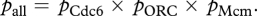


For simplicity, if we assume that all mechanisms operate with similar efficiency, so *p*_Cdc6_ = *p*_ORC_ = *p*_Mcm_ = ‘*p*_individual_’, then:
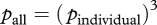
or
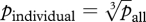
so, to achieve an overall error rate per origin of 10^−5^ needed in the budding yeast example above, then each pathway needs an error rate of 0.02. In other words, each pathway needs to be ‘only’ 98 per cent efficient.

The multiplicative relationship described above for individual mechanisms blocking re-replication suggests an interesting relationship between genome size and mechanisms preventing re-replication: addition of just one mechanism preventing re-initiation operating at approximately 99 per cent efficiency is required for every 100-fold increase in the number of origins used. Assuming origin spacing is similar, this means an additional mechanism can afford an organism a 100-fold increase in genome size.

## 6. Perspectives and challenges

The calculations described above are, by necessity, not based on any ‘real’ numbers. For example, what is the actual rate of re-initiation in wild-type cells *in vivo*? Previous plasmid loss assays in yeast have suggested that the rate of re-initiation per origin is less than 1 in 10^−3^ [96], which was the limit of detection in this assay. However, as described above, the real number is likely to be much lower than this. Similarly, just how efficient is the ubiquitin-mediated degradation of Cdc6 *in vivo*? Or, how efficient is the export of Mcm2-7? Are they 90, 99, 99.99 per cent efficient? Assays that can measure intracellular concentrations of proteins over the ranges required simply do not exist. Hopefully, the development of more sensitive, quantitative assays for these parameters will be available in the future to allow re-examination of the issues described in this review.

Finally, how important is any of this? Work in yeast has shown that mutations that decrease replication-initiation efficiency lead to greatly elevated rates of gross chromosome rearrangements [97]. Moreover, mutations that induce even small amounts of re-initiation lead to cell death [68,72] or elevated rates of gene amplification [98]. As a consequence, deregulated licensing has the potential to drive genome instability. Indeed, overexpression of Cdt1 and Cdc6 in mouse models has been shown to induce tumours [99,100]. Deregulated CDK expression is common in cancer and has been shown to inhibit licensing in both yeast and human cells [101–103]. Thus, the ability to initiate replication efficiently and, at the same time, efficiently prevent any re-initiation may be a critical barrier to the development of cancer.
